# Encouraging General Practitioners to Refer Patients With Insomnia to a Digital Therapeutic (Sleepio): Feasibility Repeated-Measures Intervention Study

**DOI:** 10.2196/75359

**Published:** 2025-08-25

**Authors:** Ohoud Alkhaldi, Brian McMillan, John Ainsworth

**Affiliations:** 1 Division of Informatics, Imaging and Data Sciences, School of Health Sciences, Faculty of Biology, Medicine and Health, University of Manchester, Vaughan House, Portsmouth St, Manchester, M13 9GB, United Kingdom, 44 7415085174; 2 Centre for Primary Care and Health Services Research, Division of Population Health, Health Services Research and Primary Care, School of Health Sciences, Faculty of Biology, Medicine and Health, University of Manchester, Manchester, United Kingdom; 3 National Institute of Health and Care Research (NIHR) Manchester Biomedical Research Centre, Manchester University Hospitals National Health Service (NHS) Foundation Trust, Manchester Academic Health Science Centre, Manchester, United Kingdom

**Keywords:** digital therapeutics, mobile app, insomnia, behavior change, primary care

## Abstract

**Background:**

Sleepio, a digital therapeutic offering digital cognitive behavioral therapy for insomnia, has been recommended by the National Institute for Health and Care Excellence in the United Kingdom as an alternative to offering sleep hygiene or sleeping pills. However, understanding of the referral behavior of general practitioners (GPs) regarding Sleepio is lacking.

**Objective:**

The aim of this study was to investigate the feasibility of using an intervention targeting GPs in Scotland to increase referrals of patients with insomnia to Sleepio.

**Methods:**

GPs working in primary care in Scotland were invited to join the study. GPs were recruited through the Primary Care Research Network in Scotland from June 10, 2024, to October 13, 2024. The behavior change wheel (BCW) was used to inform the design of the intervention. During the intervention, GPs reviewed an orientation on using Sleepio and received a visual reminder midway through the intervention. The primary outcome was the number of Sleepio referrals every 2 weeks over 2 months. The secondary outcome was the change in the GPs’ reported confidence level that Sleepio will be successful in reducing patients’ insomnia symptoms, and confidence in recommending Sleepio to patients.

**Results:**

Of the 23 GPs who joined the study, 16 completed all stages. Overall, 68.8% (11/16) of participants were females, and the mean age was 42 (SD 8) years. The total number of Sleepio referrals in 2 months was 96 for all 16 GPs. In the first 2 weeks of the intervention, the mean referral rate to Sleepio was 22.4% for all 16 GPs, but this rate increased to 45% by the end of week 8. A repeated measures analysis indicated there was no statistically significant difference in GPs’ referral rates across 4 data points. GPs’ reported confidence level in recommending Sleepio increased significantly (*z*=−3.436; *P*<.001), from a mean of 5.44 (SD 1.7; somewhat confident) to 8.13 (SD 2; very confident).

**Conclusions:**

This study explored the feasibility and impact of an intervention aimed at supporting GPs to refer patients with insomnia to the digital therapeutic, Sleepio. Improvements were seen in GP-reported confidence levels at recommending Sleepio. A large-scale intervention and a longer study duration could provide useful information concerning how long the intervention effect on GPs’ behavior toward Sleepio referrals might be maintained.

## Introduction

### Background

The use of mobile apps and digital technologies in the medical field has become common [1]. Today, health care professionals (HCPs) use apps for educational purposes, especially for medical students [2,3] but also for other purposes, such as the delivery of patient care [4]. For example, mobile health apps have helped patients to engage with their health care by enabling them to track their physical activities, as well as diet, sleep, and other health metrics [5,6]. These apps have empowered patients to take control of their health, with potential beneficial results, including better health outcomes and lower health care costs. For these reasons, many countries are now increasingly approving the use of mobile health apps and digital therapeutics [7,8]

A key difference between “health apps” and “digital therapeutics” is that the latter refers to evidence-based interventions that are clinically validated, often regulated, and designed to treat, manage, or prevent diseases [9].

Sleepio is a digital therapeutic using digital cognitive behavioral therapy for insomnia (CBTi) and has been recommended by the United Kingdom’s National Institute for Health and Care Excellence (NICE) for treating patients with insomnia and insomnia symptoms [10]. The clinical evidence for Sleepio indicates that it is an effective treatment for insomnia and can improve insomnia symptoms and poor sleep [11]. NICE has concluded in-year cost savings over usual insomnia treatments in primary care [10]. If a medication is thought to be required to help with trouble sleeping, a brief course of benzodiazepines or Z drugs may be provided (treatment for insomnia). However, CBTi may be preferable to some medications that may be addictive [12]. According to the guidelines, CBTi is the first-line recommended treatment for insomnia, whereas sleep medications are not [13,14]. Therefore, as part of the Scottish Government’s National Digital Mental Health Programme, Sleepio is currently available free to all adults across Scotland until 2030, through which patients can access the treatment directly by self-referral or can be signposted to it by their GPs [15].

Nevertheless, general practitioners (GPs) have been slow to adopt digital therapeutics. They have reported that some of the barriers that dissuade them from prescribing such treatments include a lack of awareness and knowledge, concerns about app quality and regulations, and limited training and support [16-18]. Consequently, several studies have been conducted to support GPs to incorporate digital therapeutics into their practices. A systematic review of interventions aimed at helping HCPs overcome barriers to prescribing digital therapeutics revealed that most interventions were successful in changing HCPs’ behavior toward app prescriptions [19]. The most commonly reported outcome measures were changes in the numbers of prescriptions, changes in the levels of knowledge about health apps, or changes in the reported level of confidence about prescribing digital therapies. Restructuring the GPs’ environment, such as offering technical resources [3,20] or providing education and training, was most likely to produce changes in HCPs’ prescribing behavior [21,22].

Similar barriers have been reported in a survey of GPs in Scotland regarding referring their patients with insomnia to Sleepio [23]. This survey study showed that GPs would recommend Sleepio to their patients if they had better knowledge regarding the evidence confirming the benefits of Sleepio. GPs predicted that they would develop a habit of referring patients to Sleepio if they knew how to determine if someone would benefit from it. This interventional study was based on this existing literature, but it adopts a theory-driven approach that incorporates the behavior change wheel (BCW) to address some of the behavioral barriers to the adoption of Sleepio [24]. The BCW is an effective framework to systematically understand the target behavior and identify the barriers and enablers of the behavior. It consists of 8 steps to guide the design of a behavior change intervention. It starts with addressing Capability, Opportunity, and Motivation sources of Behavior (COM-B) model that represents the hub of the BCW. Then, it goes through the second and outer layer of the wheel by identifying intervention functions and policy categories. Once details from the BCW have been obtained, the appropriate behavior change techniques can be identified. The application of BCW could assist in the integration of Sleepio into clinical practice, thereby improving the uptake of NICE guidance and the benefits to patients and cost savings to the National Health Service identified therein.

### Objective

This study aimed to explore the feasibility of using an intervention to support GPs to refer patients to a digital therapeutic (Sleepio) for the treatment of insomnia.

## Methods

### Study Participants

Inclusion criteria were currently practicing GPs (full time or part time) who worked in Scotland and provided informed consent. No exclusion criteria were applied. To facilitate participation, an internet-based study was conducted from June 10, 2024, to October 13, 2024. This decision was made because GPs have busy schedules, which were taken into account when designing the intervention by applying the 8 steps of the BCW. The mode of delivery was therefore chosen to be internet-based, at the individual level, and accessible via computers at a time convenient for the participants. This approach was intended to encourage GPs to participate by accommodating their workload and time constraints. Participants were recruited through the Primary Care Research Network in Scotland [25]. Interested GPs were asked to contact the research team via email.

Based on the practical considerations of recruiting viability and available resources, a target sample size of 12 participants was chosen based on recommendations in the literature for feasibility studies [26]. However, we exceeded this target and successfully recruited 16 GPs. This number was more likely to offer reliable information and enhance the robustness of the findings.

### Study Intervention

The BCW framework was used to systematically understand the target behavior and inform intervention design in terms of targeting the COM-B system [27]. Our prior research followed the 8 steps of BCW to design this intervention [23]. It involved a survey of GPs working in primary care settings, identified their attitudes toward prescribing CBTi, and analyzed the sources of behavior using the COM-B self-evaluation questionnaire. Capability was addressed by introducing Sleepio to GPs and providing them with instructions for recommending and using Sleepio. Opportunity was targeted by addressing technical issues and solving accessibility problems with Sleepio. Motivation was addressed by supporting GPs to develop habits by asking them to report the number of referrals they made every 2 weeks and by sending them visual email reminders.

A preintervention questionnaire was sent to all study participants to collect demographic information and to identify GPs’ current behavior and confidence toward referring patients to Sleepio (Multimedia Appendix 1).

At the start of the intervention, GPs were asked to review an orientation presentation on their own time regarding using the Sleepio digital therapeutic in patient care (Multimedia Appendix 2). The duration of the orientation presentation was approximately 11 minutes. The objective of the orientation was to enhance the GPs’ confidence in referring patients to Sleepio by ensuring that they understood how Sleepio treats insomnia, the clinical credibility Sleepio has as a digital treatment, and how patients can have instant access to Sleepio.

The influence of the training on each GP’s self-reported referrals to Sleepio was assessed every 2 weeks over 4 time periods (2, 4, 6, and 8 weeks) (Multimedia Appendix 3).

The participants received visual reminders to recommend Sleepio to their patients at 4 weeks after the beginning of the study (Multimedia Appendix 4). Ongoing technical support was available for the GPs to solve any technical issues during the intervention. The study period was 2 months.

### Outcome Measures

The primary outcome measure was the change in self-reported Sleepio referrals over time, at 2, 4, 6, and 8 weeks after reviewing the orientation presentation about the use of Sleepio in patient care. The number of Sleepio referrals by each GP was collected every 2 weeks via short internet-based forms using Qualtrics [18].

The secondary outcome was the change in GPs’ confidence in Sleepio as a successful treatment for insomnia and their confidence in recommending Sleepio to their patients before and after completing the intervention.

### Data Analysis

Data were analyzed using SPSS Statistics (IBM Corp). Descriptive statistics, including mean and SD or median and IQR for continuous data and percentages for categorical data, were used. The primary outcome, which is the change in Sleepio referrals at 4 data points, was analyzed using the Friedman test. Changes in Likert-scale questions about confidence before and after the intervention were analyzed using the paired *t* test (2-tailed) and Wilcoxon signed-rank test.

### Ethical Considerations

Ethics approval was obtained from the University of Manchester research committee (reference numbers 2023-18524-32221; 24/NRS/0015) and Health Research Authority (reference number 326797). All participants gave their informed consent for inclusion before they participated in the study. Compensation included £97 (US $124) for participating in the study. Data were anonymized for analysis to help preserve privacy and confidentiality. Participants were informed that participation in the research was voluntary and that they could withdraw their consent at any time without giving a reason.

## Results

### Overview

A total of 23 participants signed the consent form and joined the study. Out of 23 participants, 6 GPs dropped out before the start of the study, and another dropped out after the first data collection. Overall, 16 GPs completed the full intervention and became the participants of the statistical analysis. The mean age was 42 (SD 7.9) years, and 68.8% (11/16) of the GPs were female (Table 1). The participating GPs spent an average of 6 sessions in their practice per week. A session is the time GP spent on the provision of care [28].

**Table 1.** Demographics of general practitioners who completed the study (N=16).

Variables	Values
Gender, n (%)	
Male	5 (31.3)
Female	11 (68.8)
Age (years), mean (SD)	42.88 (7.9)
Number of sessions, mean (SD)	5.94 (1.8)
Size of the general practice, n (%)	
Small (<3000 patients)	4 (25)
Medium (3000‐10,000 patients)	10 (62.5)
Large (>10,000 patients)	2 (12.5)
Patients presented with insomnia per week, n (%)	
<5	9 (56.3)
5‐10	7 (43.8)
Prescribed digital CBTi^a^ to treat insomnia in the past 12 months, n (%)	
Daily	0 (0)
Weekly	5 (31.3)
Monthly	4 (25)
Rarely	5 (31.3)
Never	2 (12.5)
Aware that Sleepio has been recommended by NICE^b^, n (%)	
Yes	14 (87.5)
No	2 (12.5)
Any specific training on Sleepio, n (%)	
No	16 (100)

a CBTi: cognitive behavioral therapy for insomnia.

b NICE: National Institute for Health and Care Excellence.

Overall, 87% (14/16) of GPs reported that they were aware that NICE has recommended Sleepio as an alternative treatment to the usual insomnia treatments. None of the GPs had received any previous training about Sleepio.

### Referrals

**Figure 1.**
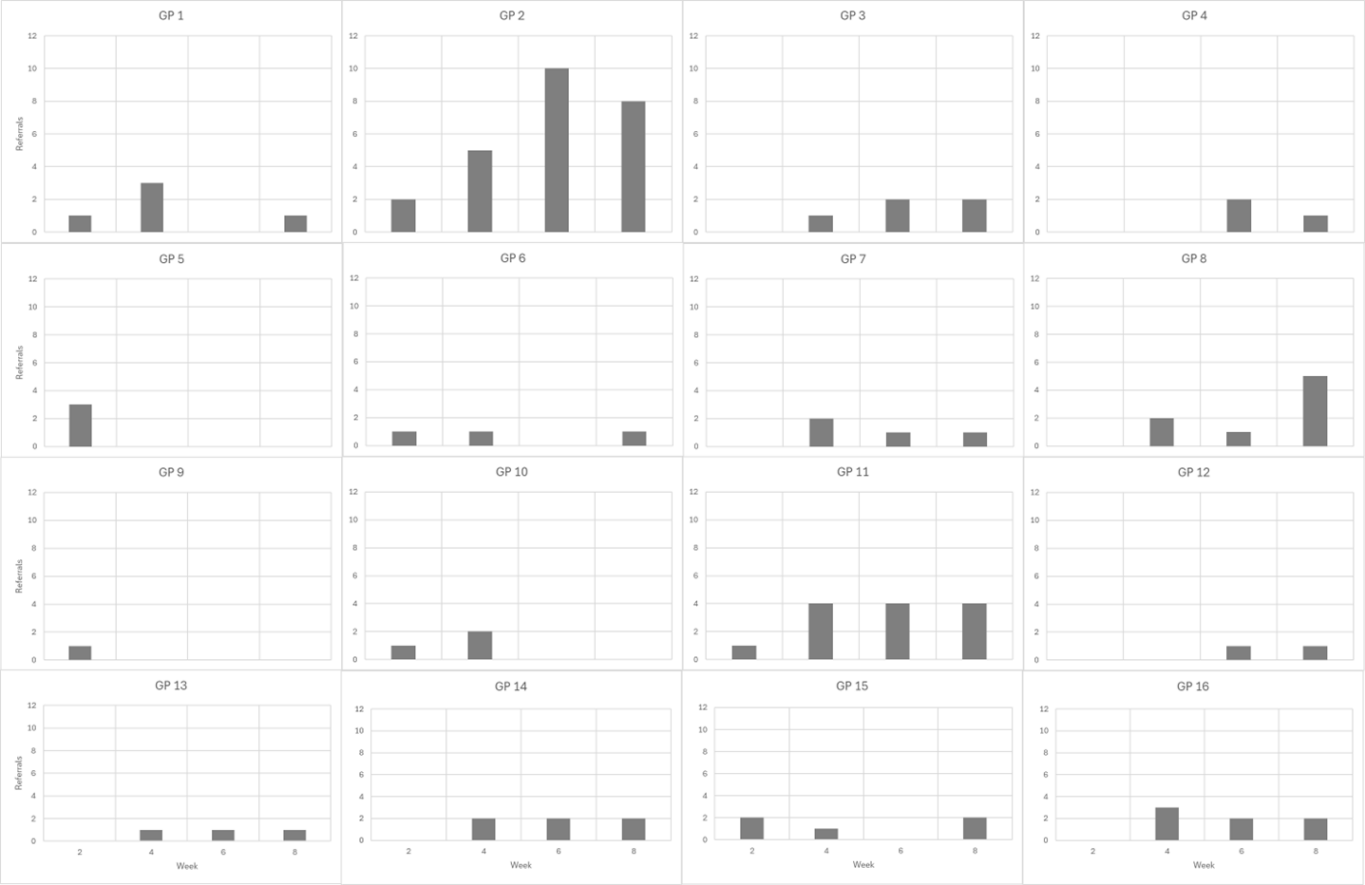
Frequency of Sleepio referral per GP. GP: general practitioner.

**Figure 2.**
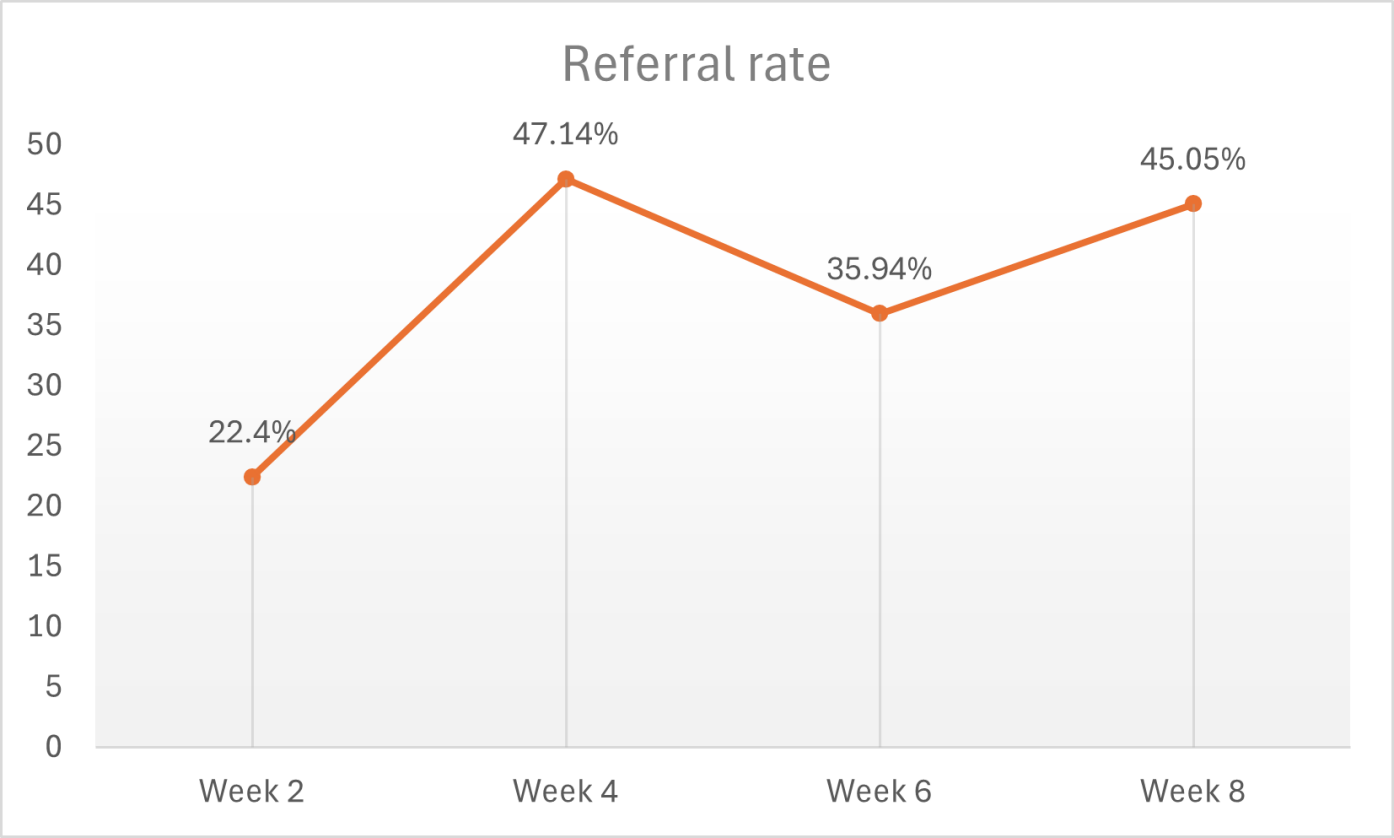
General practitioners’ referral rates to Sleepio.

The total number of referrals made by GPs over the 2-month study period was 96. The number of referrals to Sleepio per GP was determined at each data collection point (ie, at 2, 4, 6, and 8 weeks; Figure 1). The mean referral rate to Sleepio at week 2 of the intervention was 22.4%, and this doubled to 47.14% at week 4. A drop in referral rates to almost 36% was noted in week 6. The last 2 weeks of the intervention had higher Sleepio referral rates, at 45% (Figure 2).

The Friedman test was conducted to determine if any significant differences were present in the number of referrals across the 4 time points (week 2, week 4, week 6, and week 8) during the intervention. The results indicated no statistically significant differences in the referral rates across the time points (*χ*²_3_=6.024; *P*=.11). This suggests that the intervention did not result in significant changes in referral numbers over the measured weeks.

### Confidence

The GPs showed significantly improved confidence in referring patients to Sleepio (Table 2). They reported that they were confident that Sleepio was a successful treatment for reducing insomnia symptoms. To measure the change in GPs’ confidence level before and after the intervention, the paired *t* test was used. It revealed a significant increase in confidence level following the intervention, with a mean difference of 1.44 (SD 2, 95% CI 0.37-2.50; *P*=.01).

Regarding the GPs’ reported confidence in recommending Sleepio to their patients, the Wilcoxon signed-ranks test showed a significant increase in confidence levels following the intervention (*z*=−3.436; *P*<.001), with 15 participants showing improved scores and 1 showing no change.

**Table 2.** General practitioners’ confidence level before and after the intervention.

Confidence level	Baseline rating	Postintervention	*P* value
Digital CBTi^a^ (Sleepio) is successful in reducing patients’ insomnia symptoms, mean (SD)	5.06 (1.7)	6.50 (1.5)	.01
Recommending digital CBTi (Sleepio) to a patient^b^	5.44 (1.7)	8 (2)	<.001

a CBTi: cognitive behavioral therapy for insomnia.

b Baseline rating for recommending digital CBTi (Sleepio) to a patient is mean (SD); postintervention for recommending digital CBTi (Sleepio) to a patient is median (IQR).

## Discussion

### Principal Findings

The purpose of this study was to explore a feasibility intervention aimed at increasing the use of the Sleepio digital therapeutic in primary care. The GPs received orientation material at the start of the intervention about the use of Sleepio as a treatment for patients with insomnia, and they were asked to report the number of Sleepio referrals they made every 2 weeks.

Over the 2-month study, a total of 96 Sleepio referrals were made by the 16 participating GPs. This number was similar to some previously reported values [29] but lower than the number reported for other interventions in the literature [30,31]. The repeated self-reported Sleepio referrals revealed no significant differences between the 4 time point measurements. The observed variation in the fortnightly Sleepio referrals could be because of the timing during the year, as seasonal fluctuations in different factors, such as GP availability and patient consultation rates, could influence the patterns of referral to Sleepio [32]. Moreover, the behavior of patients with insomnia toward sleeping issues and barriers encountered when seeking treatment could also influence GPs’ referrals to Sleepio [33.

Nevertheless, the intervention was clearly effective in improving the GPs’ confidence in the Sleepio. This is promising, as the lack of confidence among GPs in discussing and recommending nonpharmacological treatments remains a significant barrier to the adoption of these treatments [34]. This reluctance originates from the GPs’ limited knowledge or training in CBTi but is exacerbated by accessibility issues, as some GPs may not know how to refer patients to CBTi services. Therefore, many GPs may tend to prescribe short-term pharmacological treatments for their patients with insomnia rather than endeavoring to treat the underlying behavioral causes.

The discrepancy between the significant improvement in GPs’ confidence in recommending Sleepio and a lack of statistically significant change in referral rates might be an indication of a typical time delay or lag between changes in attitudes and real behavioral changes in clinical practice. Although greater confidence is a sign that one is prepared to suggest digital CBTi, it sometimes can take more time and repeated reinforcement for behavioral changes to become completely incorporated in daily routine. In addition, it is likely that the improved confidence resulted in more suitable and selective referrals rather than increased frequency overall, especially if GPs were better able to identify which patients would benefit the most.

This study was built on our previous study that used the BCW to design a tailored intervention aimed at overcoming GPs’ perceived barriers to referring patients with insomnia to Sleepio [23]. A key objective of this design was to enhance GPs’ knowledge and confidence in making these referrals by incorporating targeted educational and practical components. The findings of this study demonstrate a significant improvement in the GPs’ confidence levels, thereby validating the strategies proposed during the design phase. This alignment between the design objectives and the intervention outcomes underscores the utility of adopting a theory-driven approach, such as the BCW, to address specific behavioral barriers^27^.

### Strengths and Limitations

This study is the first to test the feasibility of an intervention aimed at encouraging GPs in Scotland to refer patients with insomnia to Sleepio digital therapeutics. The design of the intervention was built on a behavior-based framework, which helped (1) to understand GPs’ behavior toward using and recommending Sleepio and (2) to address GPs’ reported barriers to Sleepio referrals. The study findings demonstrate the value of using the BCW to develop tailored interventions with GPs in primary care.

The limitations of this study include its small sample size and short duration, which limited the measurement of the long-term impact of the intervention. A potential Hawthorne effect may also have existed, as participating GPs may have been motivated to refer patients to Sleepio because they needed to report the number of referrals they made every 2 weeks. A follow-up assessment study to observe whether the impact of the intervention has been sustained would provide useful information.

GP recruitment and retention in interventional studies are challenging. To promote GPs’ participation in this study, incentives were provided in return for their involvement in all research activities. While this approach may have helped recruitment, it also introduced a potential source of selection bias as some participants may have been motivated more by the financial compensation rather than their interest in the research topic. Originally, the study design did not include compensation or monetary incentives for participants. However, the Primary Care Research Network required that GPs be reimbursed for their time and contributions as a condition for promoting the study through their network. As a result, compensation was added to the protocol. Researchers designing similar studies should consider alternative forms of incentives or nonmonetary compensation to minimize selection bias while still supporting GP engagement.

Another source of self-selection bias in this study is GPs’ voluntary participation. Given their busy schedules and availability, GPs were invited to join the study voluntarily. While this approach facilitated recruitment, it has introduced self-selection bias, which means that those who joined the study may differ systematically from those who did not. For future research, randomization should be used to minimize this bias.

The primary outcome measure was based on the participants’ self-reported data. Subjective measures such as these tend to have recall issues or social desirability bias. Collecting referral data from objective tools, such as the Sleepio website or a GP record system, was not possible in this study, because of the complexity of obtaining approval at the health board level.

A control group is necessary to provide a baseline for comparisons between GPs who would get the interventions and those who would not. As a result, the true impact of the intervention can be measured and compared between the 2 groups in terms of Sleepio referrals and confidence levels.

### Conclusion

This study showed the feasibility of implementing an intervention that targeted GPs and aimed to improve their patient referrals to Sleepio and their confidence in using digital tools. The current approach of addressing GP-reported barriers to using digital therapeutics by providing educational materials and sending reminders was effective in changing the GPs’ referral behavior. Future studies should endeavor to observe long-term changes in GPs’ behavior toward Sleepio. The inclusion of patients to assess their perspective would also be beneficial.

## Supplementary material

10.2196/75359 Multimedia Appendix 1 Preintervention questionnaire assessing general practitioner demographics, insomnia management, and experience with Sleepio.

10.2196/75359 Multimedia Appendix 2 Orientation material provided to general practitioners introducing Sleepio and its use in clinical practice.

10.2196/75359 Multimedia Appendix 3 Self-report form completed every 2 weeks, recording the number of patients seen with insomnia and the number referred to Sleepio by general practitioners.

10.2196/75359 Multimedia Appendix 4 Visual reminder sent to general practitioners midway through the intervention to encourage Sleepio referrals.

## Abbreviations

BCW: behavior change wheel
CBTi: cognitive behavioral therapy for insomnia
COM-B: Capability, Opportunity, and Motivation sources of Behavior
GP: general practitioner
HCP: health care professional
NICE: National Institute for Health and Care Excellence
