# Supplementary material for: Encouraging General Practitioners to Refer Patients With Insomnia to a Digital Therapeutic (Sleepio): Feasibility Repeated-Measures Intervention Study
Source: JMIR Form Res. 2025 Aug 25;9:e75359. doi: 10.2196/75359 (PMC12377788; doi:10.2196/75359)
Supplement: Multimedia Appendix 1 [file formative-v9-e75359-s001.docx]

**Pre-intervention questions**

1. What is your gender?

- Male
- Female
- Prefer not to say
- Prefer to self-describe as ____________ (please specify)

1. Your age in years: -------------
2. How many sessions per week on average do you spend in your clinical practice?

------------------------------------------------------------------------------------------------------------------------------

1. How would you best describe the size of the general practice in which you currently work?

- Small (<3000 patients)
- Medium (3,000 – 10,000 patients)
- Large (>10,000 patients)

1. On average, how many patients present to you with insomnia per week?

- None
- <5
- 5-10
- 10-20
- >20

1. In the past 12 months, how often have you prescribed digital Cognitive Behavioural Therapy to treat insomnia?

- Once a day
- More than once a day
- Once a week
- More than once a week
- Once a month
- More than once a month
- Rarely
- Never

1. Were you aware before starting this questionnaire that the digital therapeutic (Sleepio) has been recommended by NICE as an option for treating insomnia?

- Yes
- No

1. Have you completed any specific training on Sleepio?

- Yes
- No

1. How confident are you that digital CBTi (Sleepio) will be successful in reducing your patients’ insomnia symptoms?

| 1 | 2 | 3 | 4 | 5 | 6 | 7 | 8 | 9 |
| --- | --- | --- | --- | --- | --- | --- | --- | --- |
| not at all confident | | | somewhat confident | | | very confident | | |

1. How confident would you be in recommending digital CBTi (Sleepio) to a patient?

| 1 | 2 | 3 | 4 | 5 | 6 | 7 | 8 | 9 |
| --- | --- | --- | --- | --- | --- | --- | --- | --- |
| not at all confident | | | somewhat confident | | | very confident | | |
