# Supplementary figures and images for: Encouraging General Practitioners to Refer Patients With Insomnia to a Digital Therapeutic (Sleepio): Feasibility Repeated-Measures Intervention Study
Source: JMIR Form Res. 2025 Aug 25;9:e75359. doi: 10.2196/75359 (PMC12377788; doi:10.2196/75359)

Sleepio Orientation Material


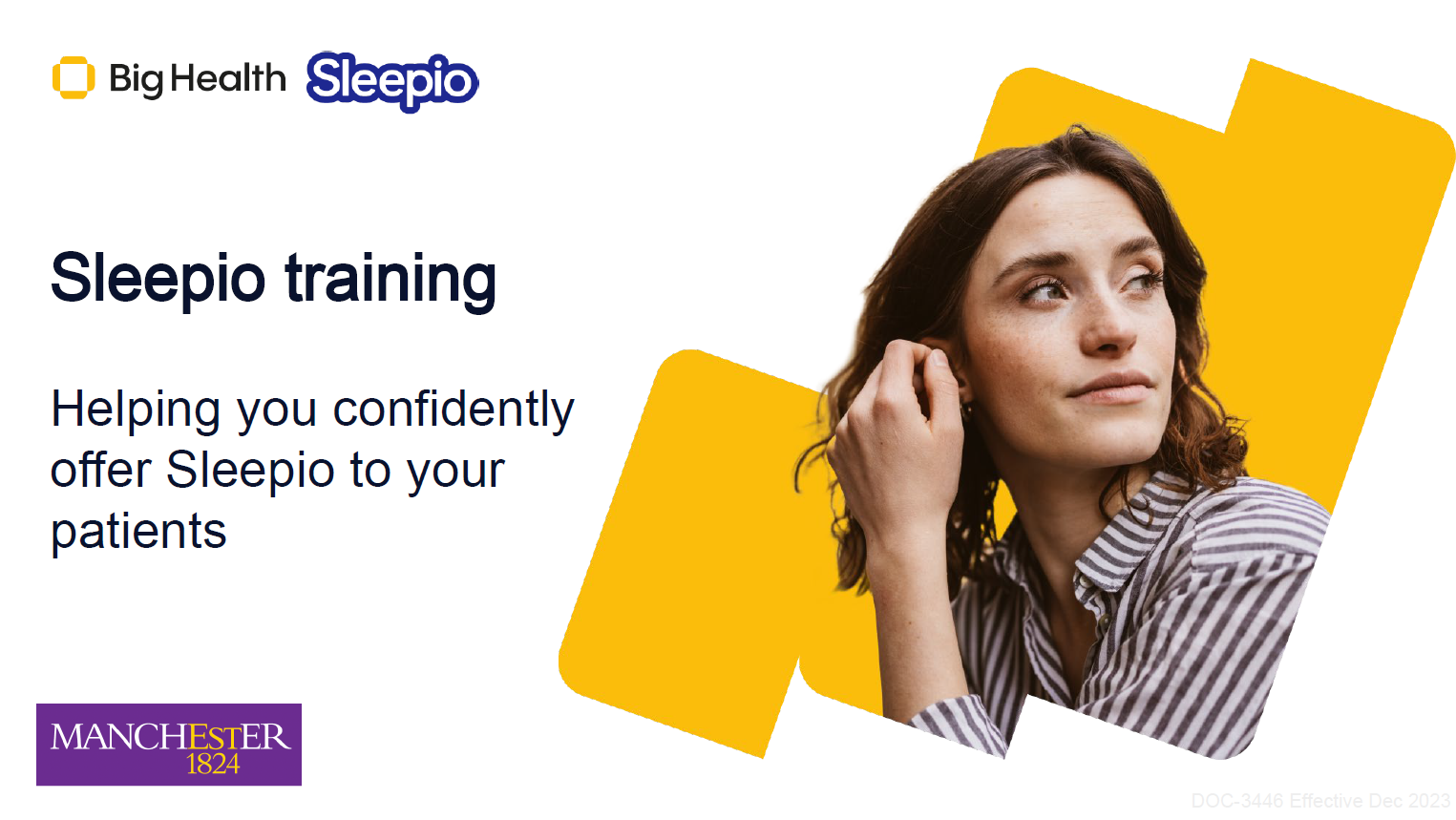

Supplement: Multimedia Appendix 2 [file formative-v9-e75359-s002.docx]

Visual Reminder


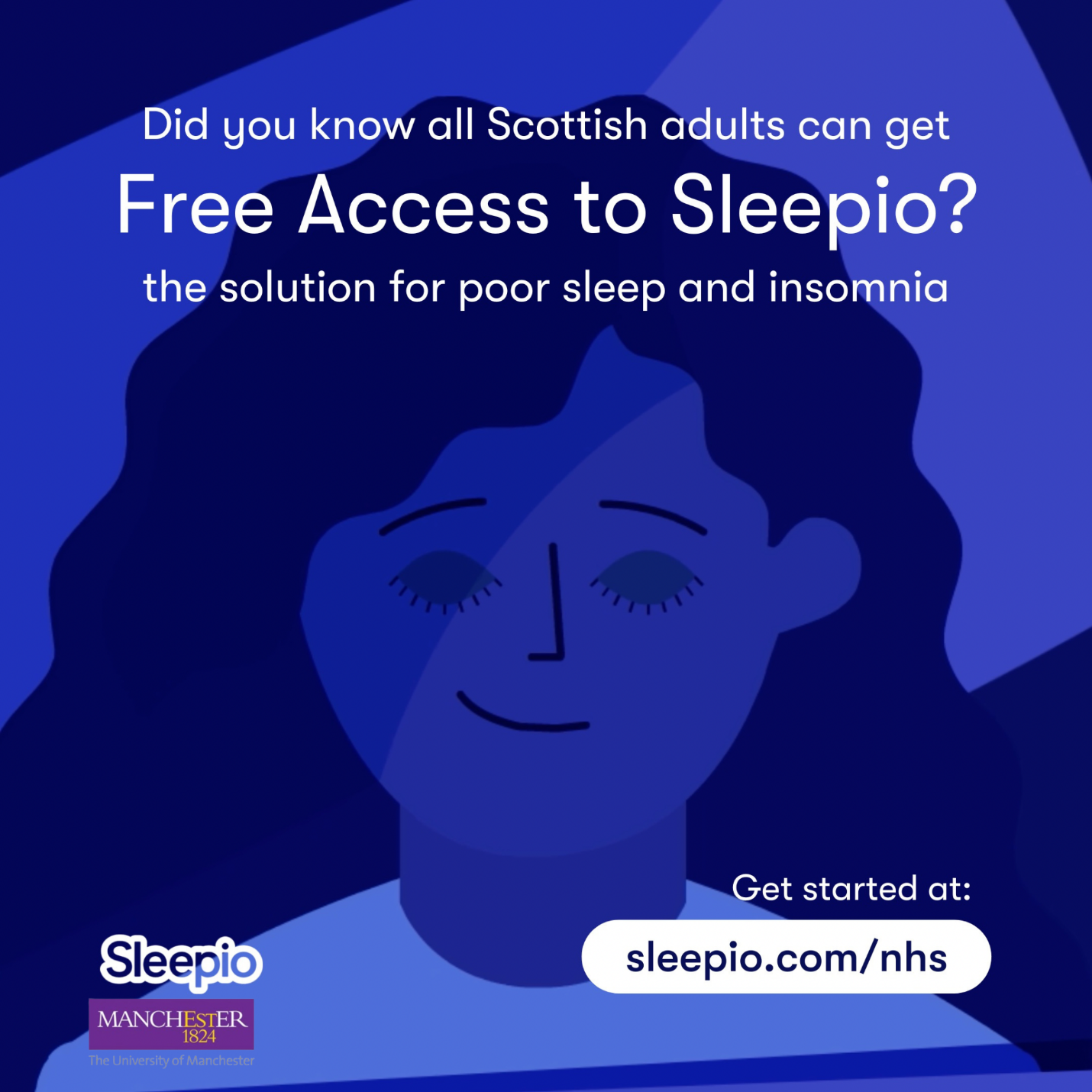

Supplement: Multimedia Appendix 4 [file formative-v9-e75359-s004.docx]
