# Supplementary material for: Encouraging General Practitioners to Refer Patients With Insomnia to a Digital Therapeutic (Sleepio): Feasibility Repeated-Measures Intervention Study
Source: JMIR Form Res. 2025 Aug 25;9:e75359. doi: 10.2196/75359 (PMC12377788; doi:10.2196/75359)
Supplement: Multimedia Appendix 3 [file formative-v9-e75359-s003.docx]

**Self-reported Sleepio Referrals (Every two weeks)**

1. How many patients presented to you with insomnia in the past two weeks?

- None
- <5
- 5-10
- 10-20
- >20

1. How many times in the past two weeks have *you* referred an insomnia patient to digital CBTi (Sleepio)?

----------------------------------------------------
